# Thoracoscopic approach for massive thymic hyperplasia in an infant: Case report and literature review

**DOI:** 10.3389/fped.2023.1144384

**Published:** 2023-03-01

**Authors:** Jinghua Jiao, Jie Yu, Chenghao Chen, Tian Chen, Tiehua Zheng, Lejian He, Qi Zeng

**Affiliations:** ^1^ Department of Thoracic Surgery, Beijing Children’s Hospital, Capital Medical University, National Center for Children’s Health, Beijing, China; ^2^ Department of Anesthesiology, Beijing Children’s Hospital, Capital Medical University, National Center for Children’s Health, Beijing, China; ^3^ Department of Pathology, Beijing Children’s Hospital, Capital Medical University, National Center for Children’s Health, Beijing, China

**Keywords:** true thymic hyperplasia, massive thymic hyperplasia, thoracoscopy, infant, thymectomy, minimally invasive surgery

## Abstract

**Introduction:**

Massive thymic hyperplasia (MTH) is a very rare entity, with fewer than 20 cases reported in the literature in infancy. Most patients have respiratory symptoms and the enlarged thymus gland occupies one side of the thoracic cavity. Posterolateral thoracotomy or median sternotomy is the main treatment for MTH in infants. We report a case of an infant with MTH in which the enlarged thymus occupied his bilateral thoracic cavity and he underwent video-assisted thoracoscopic surgery (VATS). In addition, we reviewed and summarized the relevant literature.

**Case Report:**

A 4-month-old boy was admitted to the hospital with no apparent cause of dyspnea for 18 days, with cough and sputum. On examination, the patient was found to have cyanotic lips, diminished breath sounds in both lungs, and a positive three concave sign. There was no fever or ptosis. Preoperative imaging showed large soft tissue shadows in the bilateral thoracic cavity, with basic symmetry between the right and left sides. Tumor markers were within the normal range. Ultrasound-guided fine needle biopsy showed normal thymic structures with no evidence of malignancy. As his symptoms worsened, he eventually underwent unilateral thoracic approach video-assisted thoracoscopic exploratory surgery, during which a large mass occupying the bilateral thoracic cavity was removed in a separate block and part of the thymus in the left lobe was preserved. Pathological examination confirmed true thymic hyperplasia (TTH). No relevant complications occurred at the 2-month postoperative follow-up.

**Conclusion:**

In infants, MTH occupying the bilateral thoracic cavity can produce severe respiratory and circulatory symptoms due to occupying effects. Although a definitive preoperative diagnosis is sometimes difficult, after combining computed tomography (CT) and fine needle biopsy to exclude evidence of other malignancies, the enlarged thymus occupying the bilateral thoracic cavity can be resected *via* VATS. Whether the enlarged thymus occupies the bilateral thoracic cavity and the size of the thymus are not absolute contraindications to thoracoscopic surgery. The method is safe, feasible, and minimally invasive to the patient.

## Introduction

Thymic hyperplasia is divided into two categories: true thymic hyperplasia and lymphatic follicular hyperplasia (FLH). MTH is a very rare subtype of TTH (1). The proportion of their glands far exceeds that of normal glands or the hyperplastic response to stress, as evidenced by a marked increase in the size and weight of the thymus that maintains a normal microscopic and immunohistochemical appearance (1, 2). Most patients have respiratory symptoms and the enlarged thymus occupies a unilateral thoracic cavity. Definitive diagnosis relies on fine needle biopsy or pathological examination (2–4). Surgical resection is the main treatment for MTH (5). The main surgical procedures are VATS, posterolateral thoracotomy, and median sternotomy (5,6). We report a case of VATS for massive thymic hyperplasia in an infant with enlarged thymus occupying his bilateral thoracic cavity. In addition, we discuss the clinical features, diagnosis, and treatment of this rare disease based on a comprehensive literature review and our experience.

## Case report

**Figure 1:**
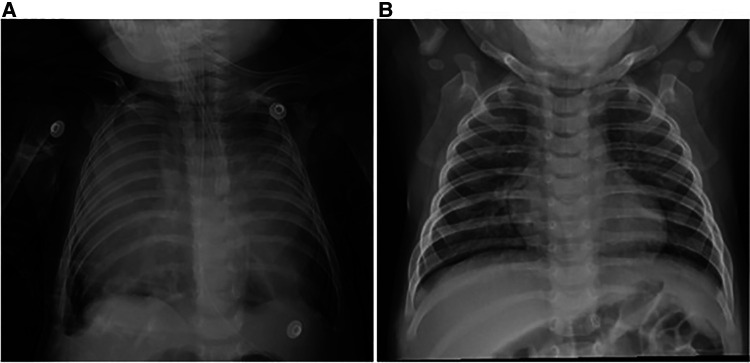
Chest x-ray. (**A**) Large soft tissue shadow in bilateral thoracic cavity. (**B**) Chest x-ray 2 months after thymectomy showed good expansion of both lungs and no recurrence.

A 4-month-old boy, weighing 6.3 kg, presented to the local hospital 18 days before the consultation with no apparent cause of dyspnea, cough, and sputum. Imaging revealed an anterior mediastinal occupancy, possible abnormal enlargement of the thymus, and bilateral pneumonia. No improvement was seen after antibiotic treatment was given. To clarify the etiology, an ultrasound-guided fine needle biopsy was performed, which showed normal thymic tissue with no evidence of malignancy. The patient was seen at our hospital for a significant worsening of symptoms, and the parents denied any other previous symptoms or comorbidities of the patient. On admission, body temperature was 37.1°C, blood pressure was 93/51 mmHg, pulse rate was 147 beats/min, and respiratory rate was 35 breaths/min. He had cyanotic lips, diminished breath sounds in both lungs, positive three concave sign, no fever, and ptosis. The peripheral blood leukocyte count was 10.38 × 10^9^/L, with 12.8% neutrophils and 81% lymphocytes. Blood gas analysis data were pH 7.403, pCO2 40.7 mmHg, pO2 51.8 mmHg, ctHb 13.9 g/dl, sO2 85.4%, BE 0.7 mmol/L, cHCO3- 24.7 mmol/L. The ultrasound showed a significantly enlarged thymus with a soft texture and fair echogenicity. Chest radiographs showed large soft tissue shadows in the bilateral thoracic cavity (Figure 1A).

**Figure 2:**
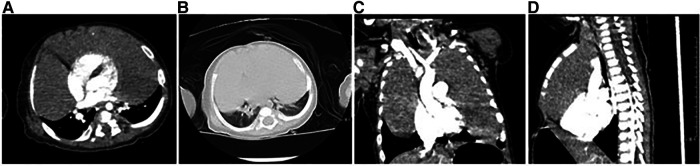
Enhanced computed tomography. (**A**) The mediastinal window shows a solid low-density soft tissue mass in the mediastinum and bilateral thoracic cavity with mild homogeneous enhancement, and the adjacent lung tissue is displaced by compression, but no involvement is seen. (**B**) The lung windows showed a decrease in volume of both lungs with no significant abnormal density shadow. (**C,D**) Bilateral soft tissue masses in the thorax were seen in the coronal and sagittal planes, growing around the heart shadow, clearly demarcated from the lung tissue, and no obvious involvement was seen.

After admission, he received non-invasive ventilation, which relieved his breathing difficulties to some extent. Enhanced computed tomography scan of the chest revealed that the thymus was significantly enlarged, it had smooth margins, regular morphology, and mild uniform enhancement, both lungs were compressed, solid, and non-distended, and the distal segments of the left and right main bronchi and their branches were thinned, and the superior vena cava, right subclavian vein, and left cephalic brachial vein were thickened, which was considered as thymic hyperplasia (Figures 2A–D). Further, the tumor markers alpha-fetoprotein (AFP), carcinoembryonic antigen (CEA), neuron-specific enolase (NSE), and human chorionic gonadotropin (HCG) in peripheral blood were checked within normal limits. Combining the patient's clinical features, imaging, and biopsy findings, TTH was considered. On the third day of admission, the patient's dyspnea further worsened and he underwent tracheal intubation. To relieve the compression of the lungs and heart and great vessels, exploratory thoracoscopic surgery was performed on the third day of admission.

The patient entered the operating room with a tracheal tube, awake and sedated, with pulse oximetry of 85%, a blood pressure of 55/40 mmHg, a heart rate of 101 beats/min, and an airway pressure of 26 cm H2O. After induction of anesthesia, the tracheal tube was removed and an Arndt5F bronchial blocker was placed in the right bronchus, followed by the insertion of a normal tracheal tube. To prevent airway collapse, muscle relaxants were not administered during anesthesia. Due to the patient's low blood pressure and slow heart rate, intraoperative blood pressure was maintained at 55–80/35–52 mmHg after pumping a small dose of dobutamine, and the heart rate fluctuated from 98 to 142 beats/min. Intraoperative blood oxygen fluctuated from 70%–100%.

**Figure 3:**
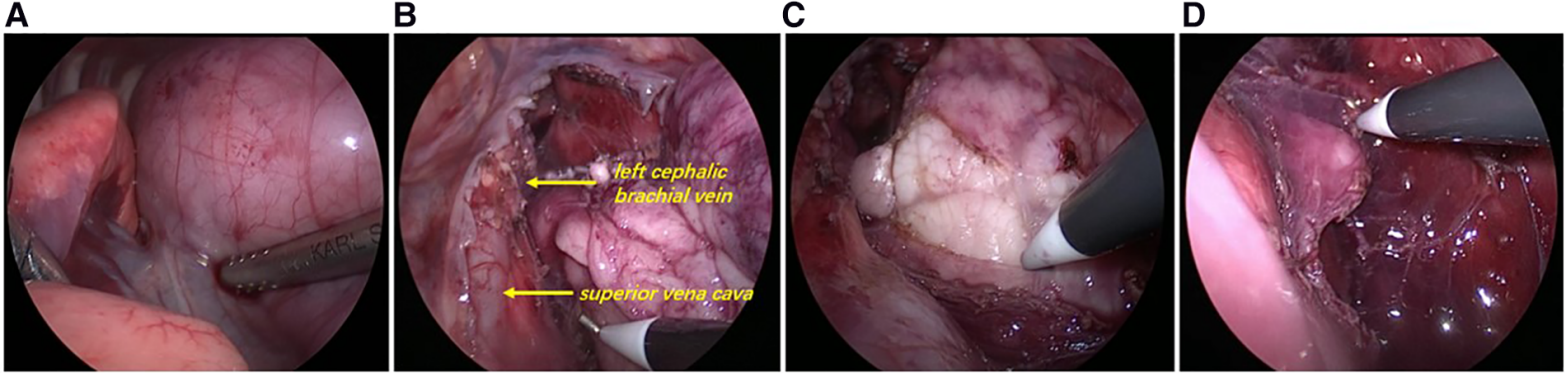
Intraoperative exploration. (**A**) Thoracoscopic exploration revealed a large mass from the mediastinum filling most of the bilateral thoracic cavity, squeezing both lungs backwards and upwards and compressing the pericardium. (**B**) The thymus wraps and compresses mediastinal structures (superior vena cava, left cephalic brachial vein, etc.), but does not attach to or invade mediastinal structures and can be easily dissected free. (**C**) Left lobe thymus was exposed during the surgery. (**D**) Part of the left lobe thymus was preserved.

**Figure 4:**
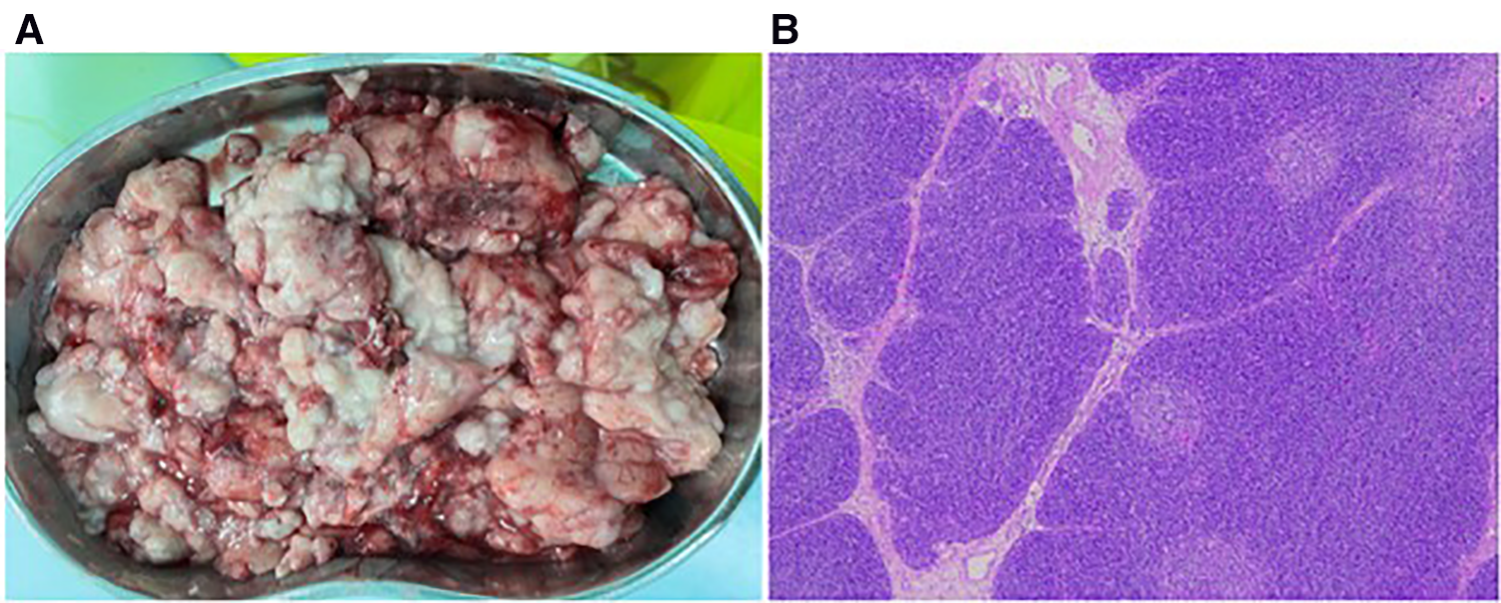
Pathological specimens and microscopic findings. (**A**) Crushed lumpy tissue with soft, homogeneous, oily feel of ash powder in the profile. (**B**) Postoperative pathological examination showed enlarged lobular structures with clear corticomedullary demarcation and visible thymic vesicles (H&E, 5×).

The patient was placed in the left lateral recumbent position and a 5 mm Trocar was placed in the 8th intercostal space/midaxillary line, 5th intercostal space/anterior axillary line, and 6th intercostal space/posterior axillary line, respectively. Artificial pneumothorax was established at 6–8 mmHg pressure. We observed giant thymus-like tissue occupying the bilateral thoracic cavity, up to the roof of the pleura and down to the diaphragm. It had an intact envelope, a yellowish-white solid lobulated shape, and a soft texture, and these were consistent with the normal thymus appearance (Figure 3A). It compressed but did not invade the surrounding structures and measured approximately 12 × 10 × 5 cm (Figure 3B), with the phrenic nerve traveling at its margin. We used an electric scalpel to first free dissect and removed the mass occupying the right side of the thorax in its entirety, followed by freeing and removing most of the mass occupying the left side of the thorax, preserving part of the thymus in the left lobe and the volume of the preserved thymus is about 1/3 of the normal unilateral thymus in the age cohorts (Figures 3C,D). And we carefully protected the phrenic nerves bilaterally during the operation. Finally, we enlarged one of the trocar holes to 2 cm and removed the tissue using a retrieval bag, and the weight of the resected mass was approximately 308 g (Figure 4A). We placed one silicone drainage tube, with intraoperative bleeding of approximately 2 ml and an operative time of 90 min. The patient had his drainage tube removed 5 days after surgery and was discharged 7 days after surgery. Postoperative pathological histology showed enlarged lobular structures, clear corticomedullary demarcation, and visible thymic vesicles. Combined with the immunohistochemical staining results, it was considered consistent with true thymic hyperplasia (Figure 4B). At the 2-month postoperative follow-up, the patient had no new symptoms or related complications. The routine blood test showed WBC 12.46*10^12^/L, LYMPH# 8.92*10^9^/L, LYMPH% 71.6%. No recurrence was detected on chest x-ray and both lungs were well dilated (Figure 1B).

## Discussion

The thymus is located in the anterior mediastinum and it is embryologically derived from the third and fourth pairs of pharyngeal pouches (7). Its size varies with age. At birth, it weighs 10–35 g and continues to grow until it reaches a maximum weight of 10 to −50 g at puberty. After this, it gradually shrinks to no more than 5–15 grams in the elderly (7, 8).

In 1978, Levine and Rosai (1) classified thymic hyperplasia into two categories based on gross and histological criteria. One type of hyperplasia is true hyperplasia, characterized by an increase in size and weight of the thymus in the absence of known irritant systemic stress and maintaining a normal microscopic and immunohistochemical presentation (9, 10). This type of proliferation is unknown in any other organ (5). Cellular enzymatic studies have shown a decrease in the number of mature T cells in cortical and medullary regions (11). The second type is lymphoid follicular hyperplasia, characterized by the activation of lymphoid follicles and germinal centers, which is the type typically associated with myasthenia gravis (12). MTH was first described by Sheila Moriber Katz (13) in 1977. Although there is no universally accepted definition of MTH, the following characteristics have been suggested in the literature: (1) It should be greater than the heart shadow on a posteroanterior chest radiograph. (2) The thymus should weigh several times the expected weight for the age of the patient. (3) It should represent more than 2% of the body mass (5).

The etiology of MTH is unknown (2) and most cases occur between 1 and 15 years of age, with rare cases within 1 year of age (5). There is a slight male predominance (14). Patients generally have symptoms of mediastinal compression or pulmonary infection in the form of dyspnea, fever, and cough (15). Infants are particularly susceptible to respiratory compromise because of the large proportion of thymus glands in the thoracic cavity (16). Peripheral lymphocytosis was found in some cases (5, 7, 13, 15–19). MTH needs to be differentiated from anterior mediastinal masses in children, such as lymphoid follicular hyperplasia, thymoma, lymphoma, teratoma, and thymic lipoma (20). Lymphatic follicular hyperplasia is most commonly seen in patients with myasthenia gravis. Thymoma usually develops at the age of 40–50 years and is very rare under the age of 20 years, while MTH patients are usually adolescents and children. In addition to the clinical features of the patient, information on the size, location, density, shape, relationship to other structures, and tissue characteristics of the mass as suggested by CT can also contribute to its differential diagnosis (21–24). Definitive diagnosis mostly relies on fine needle biopsy or pathological examination (2–4). The literature reports that steroids can be used to treat thymic hyperplasia, but are not effective against MTH (14, 17, 19, 25–28). Surgical resection is still the main treatment for MTH, including VATS, posterolateral thoracotomy, and median sternotomy. There are no complications after surgery (5, 6). Especially for patients with combined respiratory and circulatory symptoms or urgent conditions, surgery should be performed as early as possible to improve the patient's symptoms. MTH is a benign disease and previous cases have shown a good prognosis.

We searched the PubMed database for the keywords “Massive Thymic Hyperplasia”, “Massive thymic enlargement”, “Massive thymic enlargement”, “True thymic hyperplasia” and “Thymic hyperplasia”. A total of 15 cases of infancy were retrieved, and a review of these 15 patients and our patients was conducted. An additional excel file shows the details of these cases [see Supplementary material S1].

We finally identified 9 males and 6 females with a minimum age of 1 month and a maximum age of 11 months, with an average age of 6 months. The enlarged thymus occupied the unilateral thoracic cavity in 86% (12/14) of patients (5 on the left and 7 on the right), and 14% (2/14) occupied the bilateral thoracic cavity and was not explicitly mentioned in one patient. The maximum weight of the enlarged thymus was 3,550 g and the average was about 535 g. 93.3% (14/15) of patients presented with respiratory symptoms, 46.7% (7/15) presented with respiratory distress, and only one (1/15) patient had incidental findings of MTH on a chest radiograph. Peripheral blood lymphocytosis was observed in 81.8% (9/11) of patients and normal in 18.2% (2/11) of patients. 40% (6/15) of patients underwent preoperative fine needle biopsy. Three cases were diagnosed with TTH. Three cases showed normal thymic tissue, excluding evidence of malignant neoplasm. Two patients underwent mediastinotomy biopsy and one patient underwent intraoperative frozen section, both diagnosed with TTH. 26.7% (4/15) of the patients were initially treated with steroids, of which three were ineffective and one had an insignificant reduction in thymus volume. Finally, 93.3% (14/15) of the patients underwent thymectomy, and all were open procedures with no postoperative complications. Only one patient did not undergo surgery and was observed for one year without noticing a change in size. Thus emphasizing a very slow rate of size change that may lead to complications such as acute respiratory obstruction. 80% (13/15) of the patients were asymptomatic during the follow-up period. One patient developed hypogammaglobulinemia 2 years after surgery but was reported as healthy. A patient with a clinical diagnosis of MTH with Beckwith - Wiedemann syndrome presented at follow-up with a hepatic hemangioma, which was treated conservatively. Recurrence was observed in one patient during follow-up and was successfully treated with a second surgical resection, and the patient was asymptomatic at the 1-year postoperative follow-up.

Similar to most cases reported in the literature, our case initially presented with respiratory distress and peripheral blood lymphocytosis. After the surgery, his symptoms were effectively relieved and his peripheral blood lymphocyte count returned to normal. Again, our case underwent a fine needle biopsy showing normal thymic tissue, ruling out other malignancies. Notably, puncture of thymic cysts and thymomas should be avoided (29, 30). According to the literature, most of the hyperplastic thymus occupies the unilateral thoracic cavity and is more common on the right side and rarely occupies the bilateral thoracic cavity. In our case, the hyperplastic thymus occupied and almost filled the bilateral thoracic cavity with significant bilateral lung compression. If a patient with MTH is not symptomatic, hormone therapy may be given. The child in our reported case had more severe symptoms and therefore was not treated with hormone therapy. In addition, there are no clear indications regarding the duration and dosage of hormone therapy. A small number of patients have been reported in the literature to be treated conservatively, mainly because of incidental findings or because the children are almost asymptomatic and without acute. Considering that VATS is less damaging to the patient, our case underwent VATS *via* a unilateral thoracic approach after excluding the possibility of other malignancies. It should be noted that preoperative exclusion of other malignancies is important for thoracoscopic surgery to avoid causing tumor dissemination. We observed that the hyperplastic thymus was well distinguished from the surrounding tissue and did not invade the surrounding tissue and it was easily excised. The need for complete surgical removal of the enlarged thymus is controversial. Partial preservation of the thymus has been reported in the literature, with no recurrence at postoperative follow-up (13, 17, 18). The thymus is an important immune organ. Some studies have shown that thymectomy in pediatric patients, especially in infants less than 1 year of age, causes a decrease in peripheral blood T lymphocytes (2). To prevent the patient from lymphopenia, we preserved part of the thymus in the left lobe. Due to the large size of the enlarged thymus, was performed piecemeal resection without surgical complications.

## Conclusion

In summary, MTH is a rare disease with a favorable outcome. In infants, MTH occupying bilateral thoracic cavity is very rare and can produce severe respiratory and circulatory symptoms due to the occupying influence. Since intubation is difficult in patients with acute airway obstruction caused by an anterior mediastinal mass, surgery should be performed as soon as possible when patients develop respiratory and circulatory symptoms or recurrent infections. Although a definitive preoperative diagnosis is sometimes difficult, the combination of CT and fine needle biopsy can help rule out evidence of malignancy. Since the enlarged thymus is soft, with an intact envelope and no invasion of the surrounding tissues, the surgical option of VATS is available. We believe that whether the enlarged thymus occupies the bilateral thoracic cavity and the size of the thymus volume are not absolute contraindications to VATS. Combining our unit's previous surgical experience and those reported in the literature, the surgical approach of resection of giant thymic hyperplasia *via* VATS is safe and feasible. Therefore, we recommend that teams of experienced anesthesiologists and surgeons at tertiary care hospitals may consider VATS as an option for the treatment of MTH in infants.

## Data Availability

The original contributions presented in the study are included in the article/**Supplementary Material**, further inquiries can be directed to the corresponding author/s.

## Supplementary material

Previously reported cases of MTH in children aged <1 year of life. A table shows the details of these cases. The Supplementary Material for this article can be found online at https://www.frontiersin.org/articles/10.3389/fped.2023.1144384/full#supplementary-material.
